# Where a Nursing Sister Was Not Even a Nurse

**Published:** 1916-03-11

**Authors:** 


### THE EDITOR'S LETTER-BOX.

**Our Correspondents are reminded that brevity of style and conciseness of statements greatly facilitate early insertion. We cannot in any way be responsible for the opinions expressed by correspondents, who must give their name and address as a guarantee of good faith, but not necessarily for publication.**

### Where a Nursing Sister was not even a Nurse.

### A REMARKABLE JUDGMENT.

*To the Editor of* The Hospital.

Sir,—The following is reported in the *Daily Mail* under date March 7: "Mr. Justice Bargrave Deane, in the Probate Court yesterday, was asked to admit to probate the contents of a letter dated Oct. 8, 1915, written by Ada Stanley, who died at Netley Hospital on Dec. 23. Counsel asked that it should be regarded as a soldier's will.

"Mr. Justice Bargrave Deane: Is it the old story, can a woman be a soldier?

"Mr. Willis: Yes; but I hope we have advanced since the days of Charles II.

"His Lordship: But this lady was not even a nurse; she was a nursing sister."

The above statement comes as a surprise to one who has successively held the rank of probationer, nurse, nursing sister, matron, and lady superintendent, and had always imagined that these titles denoted that a woman was a member of the nursing profession and had gained admission to the higher ranks by faithful service in the lower. To describe anyone as being "only a nursing sister and not a nurse" seems as absurd as saying "he is only a judge, not a member of the Bar." Has the nursing profession been so overrun by those who are wearing its uniform and usurping its titles that even learned judges are misled? Would it not be well that those who are still serving in the ranks should see to it that in the future there can be no mistakes as to their status? Surely such services as those which are being rendered by the nursing profession to-day deserve recognition; and could there be a better way than to make it impossible for any unauthorised person to wear the uniform, or bear the titles which belong to those who have earned them in the legitimate way?—Yours truly,

Retired Matron.

[Our correspondent may rest assured that the establishment of the College of Nursing will secure the reforms she justly advocates. We are accustomed to look to our Judges for wisdom, and to accept their judgments. Mr. Justice Bargrave Deane's reported statement that the late Ada Stanley "*was not even a nurse; she was a nursing sister,*" reveals such amazing ignorance that a knowledgeable reader's first conclusion is that the report does the Judge less than justice. We are confident his lordship would not willingly asperse the nursing sisters, who are to the nursing profession what the non-commissioned officers are to the Army—invaluable and indispensable. We believe he will take the earliest opportunity of putting this matter right.—Ed. The Hospital.]

